# Edited by T. J. Bennett; Items

**Published:** 1888-06

**Authors:** 


### Cullings from Contemporaries.

*Edited by T. J. Bennett, M. D.*

Specialists.—Dr. H. C. Van Zandt, of Schenectady Co., N. Y., (Gaillord's Medical Journal, April), relates the following: A well-to-do lady in Schenectady, having occasion to send for a physician, is reported to have prefaced the professional interview with the statement that the eminent neurologist, Dr. A., had taken charge of her brain and nervous system; Dr. B., the celebrated physical diagnostician, had looked after her heart and lungs; Dr. C., whose book on diseases of the liver had made him famous, attended to her digestive organs; Dr. D., the distinguished gynæcologist, treated her uterine functions; Dr. E., the renowned orthopœdist, had been consulted about joints and limbs; Dr. F., the great ophthalmologist, cared for her eyes and ears; Dr. G., the leading dermatologist, prescribed far a cutaneous affection.

"Why have you sent for me?" asked the doctor; "there is nothing left but the umbilicus. As my specialty is pathology, if you will have me summoned after your death, I will gladly make a post-mortem examination."

---

Amyl Nitrite vs. Atropia.—Dr. L. G. Pedigo (*Va. Medical Monthly*, May,) successfully treated a case of Atropia poisoning with Amyl Nitrite. The patient, a man about 40 years of age, called at his physician's office for a hypodermic of Morphia. The doctor, through a mistake, administered half a grain of the Sulphate of Atrophia. In a short time the patient became stupid and unconscious; in short, all the symptoms of Belladonna poisoning were manifest. Several physicians were called in, Alcohol Strychnia, Morphia, Electricity, etc., were used to no avail. Respirations were kept up artificially. The patient was rapidly sinking. Amyl Nitrite was thought of, and tried by inhalation. The first dose gave some relief. The use of the drug was persisted in and pushed for its physiological effect. The patient recovered, the first on record from half a grain of Atropia hypodermically.

---

GOVERNMENT SHOULD AID THE MEDICAL PROFESSION.—The Federal Government has paid more money in the investigation of pleuro-pneumonia and cholera in cattle and swine than for scientific research into all the diseases to which human beings are subject. Health and life are made subordinate to property.—*Judge Nesbit, of Georgia.*

---

PAINLESS DESTRUCTION OF NAEVI.—Paint the healthy skin around the nevus, for about half an inch, with flex. collodion, then apply a four per cent. solution of corrosive sublimate or collodion over the nevus, allowing the whole to remain twelve or thirteen days. When removed, there will be nothing left but a small scab. This method of treatment is said to be painless and safe.—*British Medical Journal.*

---

FOR INDIGESTION.—F. Ex. Cascara Sagrada, in two or three drop doses, at meal time, will assist digestion more than pepsin and acids. Taken in a swallow of coffee, you will not taste the cascara.

---

PERSONALS.—Dr. G. T. Strong, of Bragwell, Texas, died June 1st, of septicæmia, as the result of a wound received from the kick of his horse. He was a member of the Knights of Honor.

Dr. R. R. Obrey, of Aubrey, Texas, was arrested June 2d, on a charge of being the accomplice in a case of infanticide. The particulars of the charge have not yet been made public.

A Good Remedy for Myalgia.—

℞ Chloral Hyd.

Gum Camphor a a ʒiv.

Mix well, until liquid, and add

Lanoline ℥i.

M. S: Rub well over painful parts.

In six hours the pains were relieved, and the patient brought under the influence of chloral.—*Subscriber N. O. Med. and Surg. Journal.*

---

A number of journals contain the suggestion that drachm doses of glycerine, injected into the bowels, act specifically in overcoming constipation. The prescription is worth trying.

---

A Cure for Tuberculosis.—Professor Suton, of Reims, (*N. Y. Med. Journal,*) after a twenty years' search for a specific for tuberculosis, advises the following:

| | |
|---|---|
| Neutral acetate of copper | 0.15 grains. |
| Crystalized phosphate of sodium | 0.75 grains. |
| Glycerine, Powdered licorice | a. a. q. s. |

This is for one pill about three times a day.

---

A Point.—Do you wish to ascertain the health of a baby, feel the condition of its buttocks. If these are firm and elastic, the baby is strong and well; if they are soft, as if they were boiled turnips in a bladder, the child is out of sorts.—*Tom. Inman, M. D.*

---

The following prayer, credited to a Texas doctor, was found on the bulletin board of the New York Polyclinic recently:

"Land of the Sunny South; land of the blithsome lizzard.
Send us one breath of Spring to warm our gizzard;
Thaw our anatomy from A to Izzard,
And melt this double-damned Dakota blizzard."—*N. O. Med. and Surg. Journal.—Doctor.*

---

Seltzer water (*N. O. Med. Journal,*) allowed to flow slowly but constantly from a siphon bottle upon a burn, is said instantly to relieve pain, and to hasten final cure.

Diagnosis with Cocaine.—Baumgarten, of Buda-Pest, calls attention to the fact that a solution of cocaine applied to syphilitic and tuberculous inflammations of the larynx, never produces blanching, as it does when brushed over a catarrhal surface.

---

The following are examples of questions and answers propounded to applicants for certificates to practice, by the Virginia State Board of Medical Examiners:

*Question.*—Describe the larynx.

*Answer.*—The larynx is composed of cartilage. The œsophogus passes through the larynx.

*Ques.*—Give dose of tartar emetic.

*Ans.*—Ten grains.

*Ques.*—Give dose of sulphate of atropia.

*Ans.*—Hypodermically, 10 grains; by mouth, 60 grains.

*Ques.*—How would you treat placenta prævia?

*Ans.*—I don't know what it is.

*Ques.*—What is the source of iodine?

*Ans.*—It is dug out of the earth in blocks, like iron.

*Ques.*—Describe dengue, or break-bone, fever.

*Ans.*—By four applicants: A fever that comes on soon after the bones are broken. By one applicant: The patient should be cautioned against moving, for fear the bones should break.—*Va. Med. Nonthly, May.*

---

Local Medication of the Spinal Cord.—Dr. J. Leonard Corning, of New York (N. Y. Med. Record, March 17,) has conducted a series of experiments on rabbits, proving that medicaments injected into the vicinity of the spinal cord, modify the functions of the cord a great deal more than when injected elsewhere, or given by the stomach.

For example, the doctor injected enough strychnia to induce general convulsions and then injected cocaine to control the convulsive action. The effects of the strychnia were not controlled in the least. Another experiment was made in the same way except the cocaine was injected deep between the spinous processes of the vertebræ. This time the culvulsive action from the strychnia was controlled.

Thus Dr. Corning concludes that cocaine, thrown into the general circulation, does not counteract the toxic effects of strychnia, but that its local effect being so much greater than its general action when injected near the cord, the reflexes are paralysed, which explains this disparity of effect.

Plastic Surgery.—Thos. Linn, M. D., writing from Paris, (Phil. Med. Times, March 15,) says M. Redard has gone M. M. Peyrat, Monod and others one better, and now successfully transplants *Chicken Skin*, while the latter gentlemen transplanted only frog skin, for the purpose of filling up large losses of substance. One of Redard's patients was a young girl who, after a very profound burn on the head, had still, eight months afterwards, almost all of her head in a state of suppuration which weakened her very much and threatened her life. After two month's time, an entire regeneration of the scalp was obtained with chicken skin. The part restored measured about seven square inches. It is claimed that chicken skin, being soft, vascular and adhesive, answers admirably for plastic purposes. It sticks on without reabsorption, and forms little epidermic islands, as it were, that extend on all sides and form new tissues that is quite different from ordinary cicatricial tissue.

The skin should be taken from quite young chickens and from under their wings. It should not be deprived of cellular tissue, but should contain no fat. The wound must be aseptic and dressed with care.

